# Cochlear Implant in Children with Congenital CMV Infection: Long-Term Results from an Italian Multicentric Study

**DOI:** 10.3390/children12070908

**Published:** 2025-07-10

**Authors:** Francesca Forli, Silvia Capobianco, Stefano Berrettini, Francesco Lazzerini, Rita Malesci, Anna Rita Fetoni, Serena Salomè, Davide Brotto, Patrizia Trevisi, Leonardo Franz, Elisabetta Genovese, Andrea Ciorba, Silvia Palma

**Affiliations:** 1 Otolaryngology, Audiology, and Phoniatrics Unit, University of Pisa, 56124 Pisa, Italy; francesca.forli@unipi.it (F.F.); silvia.capobianco@phd.unipi.it (S.C.); stefano.berrettini@unipi.it (S.B.); francesco.lazzerini@unipi.it (F.L.); 2 Hearing Implant Section, Karolinska Institutet, 14186 Stockholm, Sweden; 3 Department of Neuroscience, Reproductive Sciences and Dentistry-Audiology Section, Università degli Studi di Napoli Federico II, 80131 Naples, Italy; rita.malesci@unina.it (R.M.); annarita.fetoni@unina.it (A.R.F.); 4 Division of Neonatology, Department of Translational Medical Sciences, University of Naples Federico II, 80131 Naples, Italy; serena.salome@unina.it; 5 Section of Otorhinolaryngology-Head and Neck Surgery, Department of Neuroscience, Azienda Ospedale Università di Padova, University of Padua, 35122 Padua, Italy; davide.brotto@unipd.it (D.B.); patrizia.trevisi@unipd.it (P.T.); 6 Audiology Unit, Department of Neuroscience, University of Padova, 31100 Treviso, Italy; leonardo.franz@unipd.it; 7 Otolaryngology and Audiology Unit, University of Modena and Reggio Emilia, 41100 Modena, Italy; elisabetta.genovese@unimore.it; 8 ENT & Audiology Unit, Department of Neurosciences, University Hospital of Ferrara, 44124 Ferrara, Italy; andrea.ciorba@unife.it; 9 Audiology, Primary Care Department, AUSL of Modena, 41100 Modena, Italy

**Keywords:** congenital cytomegalovirus, cochlear implantation, hearing loss, language development, speech perception

## Abstract

**Background/Objectives**: Congenital cytomegalovirus (cCMV) infection is the most common non-genetic cause of sensorineural hearing loss (SNHL) in children. In cases of severe-to-profound SNHL, cochlear implantation (CI) is a widely used intervention, but outcomes remain variable due to possible neurodevelopmental comorbidities. This study aimed to evaluate the long-term auditory and language outcomes in children with cCMV after CI and to explore clinical and radiological predictors of post-CI performance. **Methods**: Fifty-three children with cCMV and bilateral severe-to-profound SNHL who underwent CI at five tertiary referral centers in Italy were included in the study. Auditory and language outcomes were assessed pre- and post-implantation using the Categories of Auditory Performance II (CAP-II) scale, the Nottingham 3-Level Classification, and the Bates Language Development Scale. Brain MRI abnormalities were classified according to the Alarcón classification. Correlations were explored between outcome scores and symptomatic status at birth, MRI findings, and neurodevelopmental comorbidities. **Results**: At birth, 40 children (75.5%) were symptomatic and 13 (24.5%) asymptomatic. Neurodevelopmental comorbidities were present in 19 children (35.8%). MRI was normal in 15 (28.3%), mildly abnormal in 26 (49%), and moderately to severely abnormal in 12 (22.6%). Auditory and language outcomes improved significantly post-CI (*p* < 0.001), though the outcomes varied widely. Twenty-five children (47%) reached CAP level ≥ 6, and thirteen (23%) reached Bates Level 6. Symptomatic status at birth correlated weakly with worse CAP (ρ = −0.291, *p* = 0.038) and Bates (ρ = −0.310, *p* = 0.028) scores. Higher Alarcón scores were significantly associated with neurodevelopmental comorbidities, though not directly with post-CI auditory and language outcomes. Finally, the presence of neurodevelopmental disabilities was generally associated with lower results, even if without statistical significance. **Conclusions**: CI provides substantial auditory and language benefit in children with cCMV, even in cases of severe neurodevelopmental comorbidities. MRI and developmental assessments, as well as perinatal history for clinical signs and symptoms, are helpful in guiding expectations and personalizing post-implantation support.

## 1. Introduction

Congenital cytomegalovirus (cCMV) is the most common congenital viral infection worldwide, with a prevalence ranging from 0.2% to 6% in developed countries [1]. At birth, 90% of infected newborns are asymptomatic, while 10–15% present with symptoms, including jaundice, petechiae, hepatosplenomegaly, microcephaly, sensorineural hearing loss (SNHL), and ophthalmological or neurological abnormalities such as ventriculomegaly and intracranial calcifications [2].

Hearing loss is the most common and significant sequela of cCMV, making it the leading non-genetic cause of SNHL in children. Among symptomatic infants, 20–65% develop hearing loss, whereas the proportion drops to 10–15% in asymptomatic cases [3]. Hearing loss in cCMV is highly heterogeneous, varying in severity, laterality, progression, and age of onset. It can be present at birth or develop later in life, with a mean onset age of approximately 44 months in asymptomatic cases and 33 months in symptomatic cases [4]. The deficit can be unilateral or bilateral, progressive or stable, and may fluctuate over time, thus requiring long-term audiological monitoring [5,6]. The variability in hearing loss progression and severity complicates treatment decisions, and it highlights the need for early detection and intervention to optimize language and auditory development [6,7].

While some children experience mild or moderate hearing loss, a significant proportion progresses to severe-to-profound bilateral SNHL, making them candidates for cochlear implantation (CI) [7,8,9]. However, CI outcomes in cCMV patients are more variable than in those with genetic causes of deafness, primarily due to the heterogeneity of hearing loss progression, presence of neurodevelopmental disabilities, and central nervous system involvement [7,8,10]. cCMV patients can achieve meaningful auditory and linguistic gains post-CI, but their performance tends to be less predictable than in children with GJB2-related deafness, where central auditory pathways remain unaffected [7,10,11,12,13,14,15,16]. While many children affected by cCMV infection achieve functional speech perception and oral language development, those with severe neurodevelopmental impairments or extensive MRI abnormalities tend to have lower auditory and language scores [15,17], and children with mild abnormalities often perform comparably to peers with idiopathic or genetic SNHL [7,18]. On the contrary, Lyutenski and colleagues reported that even children with severe MRI abnormalities achieved functional auditory outcomes, though speech development was more limited. Moreover, they concluded that other factors, such as cognitive delay, age at implantation, and rehabilitation therapy intensity, may modulate outcomes more than MRI severity alone [17].

Neurodevelopmental disabilities are a major factor influencing variability in CI outcomes among children with cCMV infection. Several studies have shown that the presence of cognitive delay, autism spectrum disorder, or motor impairments is associated with poorer auditory perception and limited speech development after CI, even in children with functional implants and adequate device use [11,15]. In contrast, cCMV children without neurodevelopmental impairments often show auditory and speech outcomes comparable to peers with genetic hearing loss, highlighting the importance of comprehensive neurocognitive assessment when evaluating CI candidates and predicting benefit in cCMV children [7]. In addition to hearing loss, cCMV can also lead to long-term vestibular dysfunction, which may impact balance, motor development, and spatial orientation in affected children, further complicating their neurodevelopmental trajectory [19].

The aim of the present study was to explore the long-term CI outcomes in terms of speech perception and language development in a cohort of children affected by cCMV infection, diagnosed within the first three weeks of life and followed in five different Italian university hospitals.

The second aim was to identify possible factors that could predict worse outcomes post-CI, including symptoms at birth, MRI abnormalities, and neurodevelopmental comorbidities.

## 2. Materials and Methods

### 2.1. Study Design and Participants

This is a large case series study conducted across five tertiary university hospitals in Italy, as follows: University of Pisa, University of Modena and Reggio Emilia, University of Naples “Federico II,” University of Ferrara, and University of Padua. The study was approved by the Ethics Committee of University of Naples Federico II (protocol number: 274/16, 2016).

The study included 53 children diagnosed with cCMV infection and affected by bilateral severe-to-profound SNHL who underwent CI between 2005 and 2022.

Children were identified through institutional databases and clinical records from pediatric audiology and otolaryngology services at the participating centers. The diagnosis of cCMV infection was confirmed in the neonatal period through polymerase chain reaction (PCR) testing of urine samples performed within the first three weeks of life [9,20].

Children were included in the study if they had a confirmed diagnosis of cCMV infection and bilateral severe-to-profound SNHL not adequately compensated by the use of hearing aids, as well as if they had undergone unilateral or bilateral CI with optimal array insertion. Only patients with complete clinical, audiological, and neurodevelopmental follow-up data and a minimum of two years of follow-up post-CI were considered eligible.

Children were excluded if they had hearing loss due to other known etiologies, including genetic syndromes, structural cochlear anomalies, or acquired causes of SNHL. Additional exclusion criteria included the absence of neonatal confirmation of cCMV infection or incomplete clinical or follow-up data.

All patients underwent a standardized preoperative assessment protocol that included audiological testing (auditory brainstem response-ABR, otoacoustic emissions-OAE, and behavioral audiometry, and depending on age), high-resolution computed tomography of the petrous bone, high-resolution brain and inner ear MRI, and speech and language evaluation using standardized age-appropriate tools. Brain MRI was reviewed and scored according to the Alarcón classification, which stratifies the severity of cCMV-related central nervous system abnormalities [21]. In addition, each child underwent neurodevelopmental assessment performed by pediatric neurologists or developmental specialists at each center, aimed at identifying cognitive, motor, or behavioral comorbidities prior to CI. The mean age at first CI was 2.1 ± 1.5 years, with a median age of 1.5 years.

Following CI, all children underwent regular multidisciplinary follow-up at their respective tertiary care center. Postoperative assessments were conducted at standardized intervals and included both audiological and speech-language evaluations, in accordance with national Italian and institutional rehabilitation protocols [9]. Auditory perception was assessed using the Categories of Auditory Performance (CAP II) scale [22], while language development was evaluated through the Nottingham Children’s Speech and Language Assessment [23] and the Bates Language Development Scale [24]. The most recent available follow-up evaluation, performed at least two years post-implantation, was used for outcome analysis. All assessments were conducted by experienced speech-language pathologists and audiologists trained in pediatric CI rehabilitation.

### 2.2. Alarcón Classification of Brain MRI Abnormalities

Preoperative brain MRI was reviewed and classified according to the Alarcón score, a validated system for grading the severity of cCMV-related brain abnormalities based on their prognostic relevance [21]. The classification includes four grades, as reported in Table 1. This classification was applied to all patients to allow for standardized analysis of the relationship between neuroimaging severity and post-implantation outcomes.

### 2.3. Categories of Auditory Performance (CAP) Scale

Auditory performance was assessed using the Categories of Auditory Performance II (CAP-II), a hierarchical rating scale designed to measure real-life auditory functioning in children with cochlear implants. The original CAP was introduced by Archbold and colleagues, in 1995, to assess auditory perceptual abilities from basic sound awareness to telephone use [25]. The CAP-II, developed in 2010 [22], is a revised and extended version of this scale, incorporating higher-order auditory skills to better capture the range of abilities observed in modern CI users. The scale includes ten levels, from 0 (no awareness of environmental sound) to 9 (using the telephone with an unfamiliar speaker in unpredictable contexts) (Table 2). In clinical and research settings, a CAP score of 6—understanding conversation without lipreading in everyday situations—is often considered the threshold for functional auditory performance. The CAP-II scale is widely used and validated in both prospective and retrospective outcome studies.

### 2.4. Nottingham Children’s Speech and Language Assessment

Spoken language development was assessed using a three-level classification scheme adapted from clinical practice at the Nottingham Auditory Implant Programme, commonly used for monitoring pediatric CI outcomes [23]. This pragmatic tool categorizes expressive language ability into the following three broad stages that correspond to increasing communicative complexity:

- **Level 1**: pre-verbal communication (e.g., gestures and vocalizations without word use);
- **Level 2**: emergence of single words or simple two-word utterances;
- **Level 3**: connected discourse with short phrases, emerging grammar, and simple conversation.

This framework was chosen for its suitability in retrospective multicenter analysis and its alignment with real-world clinical observations. In this study, Level 3 was considered the threshold for functional spoken language, aligning with CAP-II scores of ≥6.

### 2.5. Expressive Language Levels (Adapted from the Bates Language Development Framework)

Expressive language was evaluated using a 6-level classification adapted from the MacArthur–Bates Communicative Development Inventories (CDI), a widely validated tool for assessing early language development through parent report. In this study, the expressive language scale ranged from Level 1 (pre-linguistic communication or use of isolated gestures) to Level 6 (connected multiword utterances with emerging grammar) (Table 3). This adaptation draws from the CDI: Words and Sentences form, which includes vocabulary production, grammatical complexity, and maximum utterance length [24]. The scale is particularly suited for monitoring language growth in children with CI, whose language trajectories may differ from normative age-based expectations. As demonstrated in validation studies, the CDI provides reliable estimates of expressive vocabulary and syntactic complexity even in children older than the standardization range, provided they score below ceiling [25]. In our analysis, Level 6 was considered the threshold for functional expressive language.

### 2.6. Statistical Analysis

All analyses were performed using IBM SPSS Statistics for Windows, version 23.0 (IBM Corp., Armonk, NY, USA, 2015). Categorical variables were expressed as frequencies and percentages, while continuous and ordinal variables were reported as medians with interquartile ranges (IQR), depending on the data distribution.

Pre- and post-implantation scores on the CAP-II, Bates Language Development Scale, and Nottingham 3-Level Classification were compared using the Wilcoxon signed-rank test for paired non-parametric data. To explore associations between auditory perception and language outcomes, Spearman’s rank correlation coefficient (ρ) was used.

To investigate the impact of clinical predictors on post-implantation outcomes, non-parametric tests for independent samples were employed. In particular, the Mann–Whitney U test was used to compare outcome scores between dichotomous groups (e.g., symptomatic vs. asymptomatic at birth; presence vs. absence of neurodevelopmental comorbidities). For variables with more than two categories, such as the Alarcón MRI severity score and the specific type of neurodevelopmental comorbidity, the Kruskal–Wallis H test was applied. When appropriate, pairwise post hoc comparisons were conducted using Mann–Whitney U tests with Bonferroni-adjusted significance thresholds. In addition, the association between Alarcón score and the presence of neurodevelopmental comorbidities was tested using the chi-square test. A *p*-value < 0.05 was considered statistically significant.

## 3. Results

### 3.1. Cohort Characteristics

The study included 53 children affected by cCMV infection and bilateral severe-to-profound SNHL who underwent CI between 2005 and 2022 across five Italian tertiary centers. The median age at first cochlear implantation was 1.5 years (range: 1–8 years), and the mean follow-up duration was 9.8 ± 5.3 years (range: 2–20 years). At the time of the last evaluation, 46 children (86.8%) were 7 years of age or older, corresponding to an age at which language development is generally expected to have reached a stable stage. Among the 53 patients, 22 (41.5%) received unilateral CI, while 31 (58.5%) received bilateral CI, of which 21 were sequentially and 10 simultaneously. All patients presented with normal cochlear anatomy and underwent complete electrode array insertion.

At birth, 40 children (75.5%) were symptomatic for cCMV, while 13 (24.5%) were asymptomatic. Neurodevelopmental comorbidities were present in 19 children (35.8%), with some individuals exhibiting more than one diagnosis. These included autism spectrum disorder (*n* = 5, 9.4% of the whole cohort), global developmental delay (*n* = 7, 13.2%), ADHD or behavioral dysregulation (*n* = 3, 5.6%), neurological sequelae with psychomotor delay (*n* = 5, 9.4%), specific developmental disorders (*n* = 1, 1.9%), and global immaturity (*n* = 1, 1.9%).

All children underwent preoperative brain MRI, which was assessed and classified using the Alarcón score. Imaging was normal (score 0) in 15 patients (28.3%). Mild abnormalities (score 1), such as limited white matter changes or mild ventriculomegaly, were observed in 26 children (49%). Moderate MRI changes (score 2) were present in six cases (11.3%), while severe abnormalities (score 3), including cortical malformations, corpus callosum dysgenesis, or cerebellar hypoplasia, were found in six children (11.3%).

### 3.2. Auditory Outcomes After Cochlear Implantation

**Figure 1:**
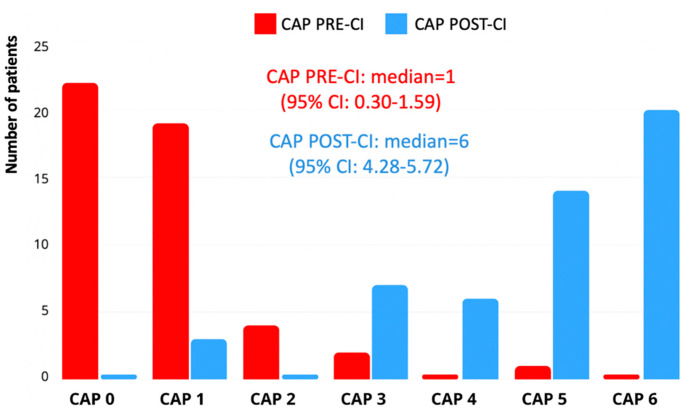
Improvement in auditory performance before and after CI assessed by CAP-II scores.

Auditory performance was evaluated using the Categories of Auditory Performance II (CAP-II) scale. Data were available for all 53 children both pre-implantation and at the most recent follow-up. The median pre-implantation CAP score was 1 (interquartile range [IQR]: 0–2), indicating limited awareness of environmental sounds or early responses to speech. At follow-up, the median post-implantation CAP score had significantly improved to 5 (IQR: 4–6), corresponding to the ability to understand conversation without lipreading with a familiar speaker. This change was statistically significant (*p* < 0.001) according to the related-samples Wilcoxon signed-ranked test. In total, 44 children (83%) reached a CAP score of ≥4, and 25 children (47%) reached CAP 6, considered the threshold for functional auditory perception (Figure 1).

### 3.3. Language Development Outcomes After Cochlear Implantation

Language development following CI was assessed using two complementary tools, as follows: Bates Language Development Scale and Nottingham 3-Level Language Classification. Overall, a significant improvement in expressive language abilities was observed across the cohort after implantation.

**Figure 2:**
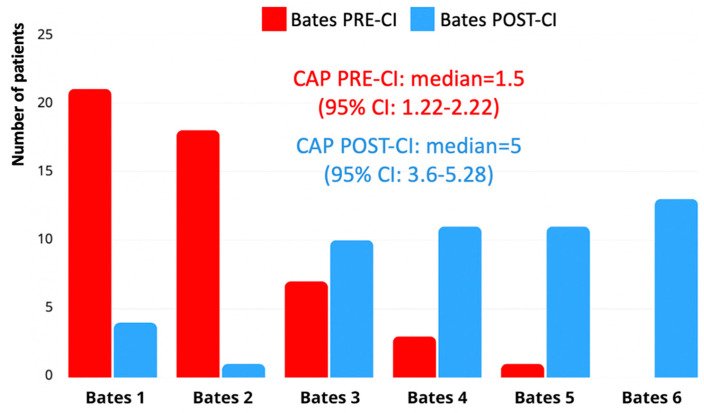
Improvement in expressive language abilities before and after CI assessed by the Bates Language Development Scale.

According to the Bates Scale, the median pre-implantation language level was 1.5 (interquartile range [IQR]: 1.22–2.22), indicating preverbal communication or early vocalizations. At the most recent follow-up, the median post-implantation score increased to a median of 5 (IQR: 3.6–5.28), corresponding to the emergence of word combinations or early sentence structures. This improvement was statistically significant (*p* < 0.001), at the related-samples Wilcoxon signed-ranked test. A total of 35 children (62.5%) reached Level 4 or higher, and 13 children (23.2%) reached Level 6, which was considered the threshold for functional expressive language (Figure 2).

**Figure 3:**
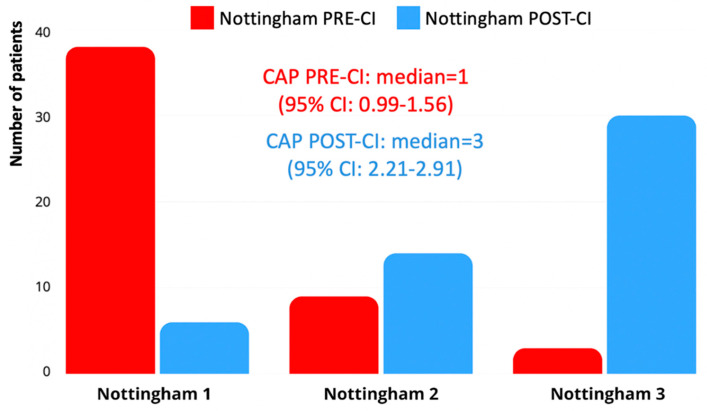
Improvement in language abilities before and after cochlear implantation assessed by the Nottingham 3-Level Language Classification.

Using the Nottingham 3-Level Language Classification, 30 children (56.6%) reached Level 3 post-CI, corresponding to connected discourse or sentence-level production. Pre-implantation, the majority of children (n = 38; 71.7%) were classified as Level 1, reflecting preverbal behavior or limited word use. Post-implantation progression was generally consistent with Bates Scale outcomes (Figure 3). These results confirm a significant enhancement in spoken language skills following cochlear implantation, although language trajectories varied considerably across individuals.

### 3.4. Correlation Analyses and Associations

Auditory perception and language development scores were strongly and significantly correlated both before and after CI. Pre-CI, CAP-II scores correlated positively with both the Bates Language Development Scale (ρ = 0.613, *p* < 0.001) and the Nottingham 3-Level Classification (ρ = 0.593, *p* < 0.001), while a strong correlation was also observed between the Bates and Nottingham scores themselves (ρ = 0.643, *p* < 0.001). These relationships were maintained post-implantation, with CAP-II showing significant positive correlations with both the Bates (ρ = 0.683, *p* < 0.001) and Nottingham (ρ = 0.686, *p* < 0.001) scores, as well as a continued strong association between Bates and Nottingham outcomes (ρ = 0.643, *p* < 0.001).

**Figure 4:**
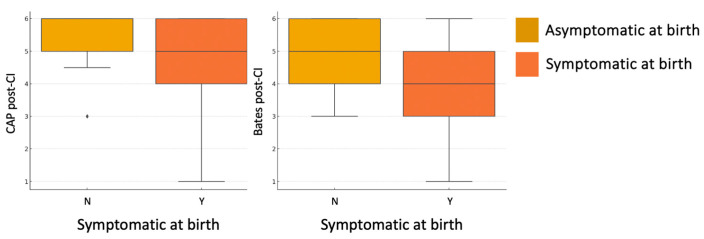
Boxplots of post-implantation CAP-II auditory scores (**left**) and Bates expressive language scores (**right**), stratified by symptomatic status at birth (asymptomatic = yellow; symptomatic = orange). Each box represents the interquartile range (25th–75th percentile), with the horizontal line indicating the median value. Whiskers extend to the minimum and maximum values within 1.5 times the interquartile range; values outside this range are shown as individual outliers. Diamond symbols indicate extreme outliers (i.e., values more than 3 times the interquartile range from the box).

A statistically significant association was found between symptomatic status at birth and both auditory and language performance post-CI. Specifically, children who were symptomatic at birth showed significantly lower CAP-II scores (U = 156, *p* = 0.040) and Bates language scores (U = 144.5, *p* = 0.030), compared to asymptomatic children (Figure 4). No significant difference was observed in the Nottingham 3-Level Classification (U = 186.5, *p* = 0.171), although the trend remained consistent.

**Figure 5:**
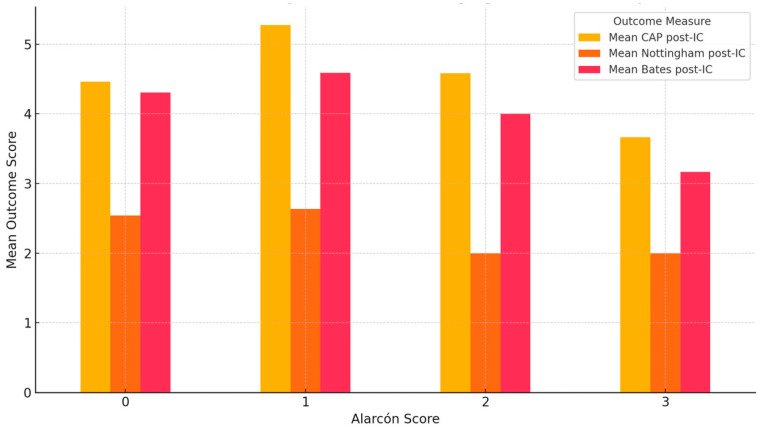
Mean post-CI auditory and language scores by Alarcón neuroimaging classification [21].

A statistically significant association was observed between Alarcón MRI scores and the presence of neurodevelopmental impairments. Specifically, children with higher Alarcón scores (2–3) were more likely to present with neurodevelopmental comorbidities compared to those with lower scores (χ^2^(3) = 14.12, *p* = 0.002). No statistically significant associations were found between the severity of brain abnormalities, as assessed by the Alarcón MRI score, and post-implantation outcomes. Specifically, Kruskal–Wallis tests revealed no significant differences across Alarcón score groups (0–3) in CAP-II scores (H = 4.283, *p* = 0.232), Bates expressive language scores (H = 1.890, *p* = 0.596), or Nottingham Language Classification (H = 4.253, *p* = 0.235). Nevertheless, children with moderate-to-severe MRI abnormalities (Alarcón scores 2–3) generally showed lower outcome scores compared with those with no or mild abnormalities, in line with previous findings, although without reaching statistical significance in this cohort (Figure 5).

**Figure 6:**
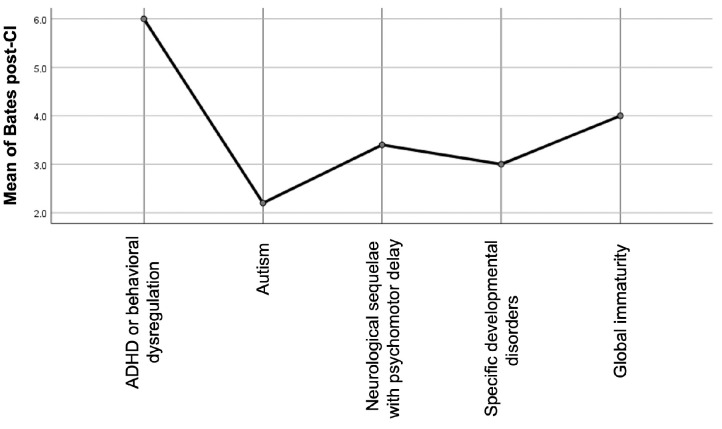
Average post-implantation expressive language scores (Bates Scale) across different neurodevelopmental comorbidity subtypes.

Similarly, no statistically significant differences were found between the overall presence of neurodevelopmental comorbidities and post-implantation outcomes when analyzed according to the specific type of condition. The Kruskal–Wallis test revealed borderline non-significant differences in both the Bates expressive language scores (H = 9.039, *p* = 0.060) and CAP-II scores (H = 9.224, *p* = 0.056), while no significant association emerged for the Nottingham classification (H = 4.659, *p* = 0.324). However, inspection of the mean ranks suggested that children with autism spectrum disorder (ASD) exhibited consistently lower post-implantation scores across all outcome measures (Figure 6). A Mann–Whitney U test comparing children with ASD to those without (including both children with other comorbidities and those without any comorbidity) confirmed a significantly lower Bates Score in the ASD group (U = 13.5, *p* = 0.043). Differences in the CAP-II (U = 17, *p* = 0.086) and Nottingham Classification (U = 21, *p* = 0.129) did not reach statistical significance but followed the same negative trend. These findings reinforce the notion that ASD may represent a particularly challenging prognostic factor in this population, even if subgroup sizes remain limited.

## 4. Discussion

This multicenter retrospective study examined auditory and language outcomes following cochlear implantation in a cohort of 53 children with cCMV infection, using a combination of auditory, speech perception, and linguistic assessment tools (CAP-II, Bates Scale, and Nottingham Classification). The results demonstrate statistically significant improvements across all outcome measures, with the majority of children reaching functional levels of auditory perception and expressive language. Although follow-up duration varied across participants, 86.8% of the cohort had reached 7 years of age or older, suggesting that most outcomes were assessed at a stage where language development is expected to be stabilized. The strong correlation between auditory and language scores both before and after implantation suggests good internal consistency among the selected tools and supports the use of complementary outcome measures to more comprehensively assess post-CI progress.

Despite the overall positive outcomes observed in our cohort, a considerable degree of variability emerged, particularly among children with neurodevelopmental comorbidities or moderate-to-severe central nervous system abnormalities on MRI. Although the presence of neurodevelopmental comorbidities did not show statistically significant differences across outcome measures when analyzed as a binary variable, children with such conditions tended to demonstrate lower scores on both auditory and language assessments. Analogous to these results, previous works have confirmed that CI offers substantial benefits in children with cCMV, albeit with greater variability than in children with genetic or idiopathic hearing loss [7,10,16,27]. Several studies have highlighted that poorer outcomes in children with cCMV tend to correlate with the presence of cognitive, behavioral, or motor impairments, rather than with the severity of hearing loss alone. Reynders and colleagues [13] emphasized that language outcomes were significantly poorer in children with additional disabilities, even when auditory thresholds were similar to peers without comorbidities. Similarly, Hoey [11] and Yoshida [10] observed that neurodevelopmental profiles, particularly involving global developmental delay and autism spectrum disorder, played a key role in limiting post-CI progress. In this study, children with autism spectrum disorder (ASD) exhibited the lowest post-implantation auditory and language scores, suggesting that ASD may be a negative prognostic factor in this population, as confirmed by Mann–Whitney U test, which showed significantly lower Bates scores in children with ASD (U = 13.5, *p* = 0.043), and similar but non-significant trends in CAP-II and Nottingham classifications. According to the review by Mathew and colleagues [28], including 28 studies and 159 children with ASD, 78% of children with ASD show some improvement in speech perception after CI, but only 63% demonstrate gains in speech expression, and a substantial proportion remain non-verbal or exhibit inconsistent device use. Similarly, studies by Palmieri [29] reported that children with multiple disabilities, including ASD, often display slower and less pronounced improvements in language and communication, but CI is still associated with notable gains in perceptual, attentional, and social domains, as reported through behavioral questionnaires.

Brain MRI findings have been proposed as a useful tool for assessing the severity of central nervous system involvement in children with cCMV and may serve as a prognostic indicator for neurodevelopmental, auditory, and language outcomes. The Alarcón score, which integrates malformative, destructive, and white matter abnormalities on the brain MRI, has been shown to correlate with the likelihood of neurodevelopmental comorbidities [21]. According to the Alarcón classification system, MRI findings can be graded from 0 to 3, with mild abnormalities (score 1), including isolated lenticulostriate vasculopathy, mild ventriculomegaly, or focal white matter signal changes, and severe abnormalities (score 3), encompassing extensive calcifications, cortical malformations (e.g., polymicrogyria), corpus callosum dysgenesis, and cerebellar hypoplasia [21]. In our cohort, a significant association was found between higher Alarcón scores and the presence of neurodevelopmental impairments, supporting the scale’s utility in identifying children at increased risk for broader developmental challenges. Though we did not observe a statistically significant correlation between Alarcón score and post-implantation auditory or language performance, patients with moderate-to-severe MRI abnormalities (Alarcón score 2–3) showed generally worse post-CI outcomes. This is consistent with the findings of Lyutenski and colleagues [17], who reported no direct relationship between MRI severity and CAP scores, despite limited language development in children with severe abnormalities. Conversely, Han [18] and Philips [7] found that children with normal or mild MRI findings performed comparably to those with genetic hearing loss, whereas severe MRI abnormalities were associated with poorer speech and language outcomes. Taken together, these data suggest that while MRI severity alone may not predict CI success, it contributes meaningfully to the overall neurodevelopmental risk profile that should inform clinical expectations and parental counseling.

In our cohort, symptomatic status at birth was weakly but significantly correlated with poorer outcomes on both the CAP-II and Bates scales. The distinction between symptomatic and asymptomatic presentation at birth in children with cCMV has long been considered a potential predictor of post-implantation outcomes. Symptomatic cCMV is typically associated with a higher risk of central nervous system involvement, including both structural brain abnormalities and neurodevelopmental impairments, which are known, in turn, to negatively affect language acquisition and auditory performance. This association was confirmed in a recent large prospective cohort study conducted by Keymeulen and colleagues [30], including 753 children affected by cCMV, which reported that 54.7% of symptomatic children developed neurodevelopmental impairments compared with only 20% in the asymptomatic group and that severe symptomatic presentations were strongly linked to abnormal brain MRI and poorer developmental outcomes. Similarly, previous studies have shown that children with asymptomatic cCMV tend to achieve better auditory and language outcomes after CI compared to symptomatic peers, with some asymptomatic children performing comparably to those with non-cCMV-related hearing loss [12,15]. However, the predictive value of symptomatic status alone remains limited, as it does not fully capture the extent of underlying neurological damage. Some children classified as asymptomatic at birth may still develop late-onset neurodevelopmental difficulties or progressive hearing loss, which can impact CI outcomes over time. These observations support the notion that symptomatic status, while informative, should be considered in conjunction with neuroimaging and developmental assessments to more accurately forecast individual trajectories and guide family counseling.

Overall, the findings of our study are essentially in line with the conclusions of a systematic review by Kraaijenga and colleagues [27], which critically evaluated CI performance in children with cCMV compared with those with other etiologies of deafness. Their review of 12 studies revealed that in 7 of them, children with cCMV had significantly worse auditory and language outcomes post-CI than non-cCMV controls, with six attributing these differences to the presence of cCMV-related comorbidities, particularly cognitive and neurodevelopmental impairments. However, when analyzing only asymptomatic cCMV children, outcomes were found to be comparable to those of non-cCMV children, reinforcing the idea that it is not the viral etiology, per se, but the associated neurological sequelae that most affect post-CI success. These results support our own data, where children with moderate-to-severe MRI abnormalities and neurodevelopmental comorbidities achieved lower scores on auditory and language scales post-CI. Moreover, the review highlighted that differences in performance tended to diminish over time, with several studies reporting convergence of outcomes after two or more years post-implantation, suggesting that CI can provide long-term benefit even in more complex cases, a conclusion also consistent with our cohort’s overall positive auditory and language progression.

This study provides additional evidence for the benefits of CI in children with cCMV, even in the presence of neurological or developmental comorbidities. Nevertheless, the variability in outcomes observed highlights the importance of multidisciplinary preoperative assessment, including neuroimaging and developmental evaluation, to guide counseling and postoperative planning. The presence of MRI abnormalities or neurodevelopmental comorbidities should not be viewed as contraindications to implantation but rather as factors that may require more tailored expectations, early intervention, and long-term speech and language support. The strong correlations between different outcome measures further underscore the need for a comprehensive and multidimensional evaluation of CI benefit, beyond auditory thresholds alone.

This study represents one of the largest multicenter cohorts to date examining CI outcomes in children with cCMV infection, with standardized post-implantation evaluation using perceptual and language measures. While its retrospective design and heterogeneity in follow-up duration and rehabilitation approaches may limit the generalizability of the findings, the large sample size strengthens the reliability of the trends observed. Future prospective studies with standardized post-CI rehabilitation protocols, consistent neurodevelopmental assessment tools, and longer follow-up into adolescence are needed to further define outcome trajectories and optimize individualized care. In particular, identifying early markers that predict language acquisition and functional communication in daily life remains a key priority in this complex population.

In this context, we finally emphasize the importance of early diagnosis of cCMV infection, via neonatal screening, which is the only tool that allows for early intervention and personalized treatment for these children. This represents one of the key factors in achieving satisfactory outcomes from the CI.

### Limitations

This study has some limitations that should be acknowledged. First, its retrospective design may introduce selection biases and limits control over some confounding variables. Second, the follow-up duration varied considerably among participants (ranging from 2 to 20 years), which could affect post-CI outcomes, particularly for younger children who may still be in active phases of language development. The lack of the ASSR test can be another limit of the study. Moreover, whether implantation was unilateral or bilateral may represent an additional variable influencing outcomes. Since the children underwent cochlear implantation over a broad time span, details such as implant manufacturer, electrode type, and processor technology varied considerably and were not systematically collected for this study. These technical aspects will be the focus of a future investigation, particularly in light of emerging evidence suggesting that different device characteristics may influence post-implantation outcomes. Information regarding the type and intensity of aural rehabilitation programs followed by each child was not available. Additionally, the lack of standardized rehabilitation protocols across centers may have contributed to inter-individual variability.

Finally, while the sample size is one of the largest reported to date for cCMV children undergoing CI, subgroup analyses (e.g., according to neurodevelopmental comorbidities) may still be underpowered to detect subtle differences.

## 5. Conclusions

CI offers clear auditory and language benefits in children with cCMV, even in the context of neurodevelopmental complexity. While outcomes are more variable than in genetic forms of deafness, most children in this large multicenter cohort achieved meaningful improvements. Preoperative brain MRI and developmental assessments remain essential tools for anticipating individual trajectories and supporting families with realistic, personalized expectations. Moreover, perinatal clinical picture may be, to some extent, considered in the prognostic assessment of these children.

## Figures and Tables

**Table 1:** Brain MRI severity classification for children with congenital CMV. Categories are derived from commonly described imaging patterns and organized progressively based on the extent and nature of central nervous system involvement, inspired by prior prognostic classifications (adapted from [21]).

Grade	MRI Abnormalities
**Grade 0**	No structural abnormalities.
**Grade 1**	Minor findings such as mild ventriculomegaly, isolated periventricular calcifications, or small focal white matter changes.
**Grade 2**	More extensive white matter involvement, presence of multiple calcifications, germinolytic cysts, or temporal lobe anomalies.
**Grade 3**	Major malformations including cortical dysplasia (e.g., polymicrogyria), callosal agenesis or hypoplasia, cerebellar underdevelopment, or diffuse cerebral atrophy.

**Table 2:** Categories of Auditory Performance (CAP-II) scale [22].

CAP-II Score	Description
**0**	No awareness of environmental sounds
**1**	Awareness of environmental sounds
**2**	Response to speech sounds
**3**	Recognition of environmental sounds
**4**	Understands common phrases without lipreading
**5**	Understands conversation without lipreading with a familiar speaker
**Functional level**	
**6**	Understands conversation without lipreading in everyday situations
**7**	Can use the telephone with a familiar speaker
**8**	Follows group conversation in a noisy or reverberant environment (e.g., classroom)
**9**	Uses the telephone with an unfamiliar speaker in unpredictable situations

**Table 3:** Expressive language development scale for children with hearing loss. This 6-level progression describes typical stages of early expressive language observed in clinical settings and draws conceptual reference from the MacArthur–Bates framework (adapted from [26]).

Language Level	Description
**Level 1—Prelinguistic**	Use of gestures, vocalizations, and babbling without consistent word use.
**Level 2—Single Words**	Occasional use of isolated meaningful words; limited vocabulary.
**Level 3—Word Combinations**	Emergence of two-word phrases or short utterances
**Level 4—Early Sentences**	Use of short but structured sentences; basic grammar elements appear.
**Level 5—Sentence Expansion**	Increasing complexity of sentence structure and vocabulary.
**Level 6—Connected Language**	Fluent use of language in full sentences, appropriate for context and conversation.

## Data Availability

Data for the current study are not available due to privacy restrictions.
